# *Arabidopsis* G-Protein β Subunit AGB1 Interacts with BES1 to Regulate Brassinosteroid Signaling and Cell Elongation

**DOI:** 10.3389/fpls.2017.02225

**Published:** 2018-01-09

**Authors:** Ting Zhang, Pengbo Xu, Wenxiu Wang, Sheng Wang, Julie C. Caruana, Hong-Quan Yang, Hongli Lian

**Affiliations:** ^1^School of Life Sciences and Biotechnology, Shanghai Jiao Tong University, Shanghai, China; ^2^State Key Laboratory of Genetic Engineering and Collaborative Innovation Center for Genetics and Development, Institute of Plant Biology, School of Life Sciences, Fudan University, Shanghai, China; ^3^Department of Cell Biology and Molecular Genetics, University of Maryland, College Park, MD, United States; ^4^Key Laboratory of Urban Agriculture (South), School of Agriculture and Biology, Shanghai Jiao Tong University, Shanghai, China

**Keywords:** BR signaling pathway, G-protein signaling pathway, BES1, AGB1, protein interaction, phosphorylation status, transcription activity

## Abstract

In *Arabidopsis*, brassinosteroids (BR) are major growth-promoting hormones, which integrate with the heterotrimeric guanine nucleotide-binding protein (G-protein) signals and cooperatively modulate cell division and elongation. However, the mechanisms of interaction between BR and G-protein are not well understood. Here, we show that the G-protein β subunit AGB1 directly interacts with the BR transcription factor BES1 *in vitro* and *in vivo*. An AGB1-null mutant, *agb1-2*, displays BR hyposensitivity and brassinazole (BRZ, BR biosynthesis inhibitor) hypersensitivity, which suggests that AGB1 positively mediates the BR signaling pathway. Moreover, we demonstrate that AGB1 synergistically regulates expression of BES1 target genes, including the BR biosynthesis genes *CPD* and *DWF4* and the *SAUR* family genes required for promoting cell elongation. Further, Western blot analysis of BES1 phosphorylation states indicates that the interaction between AGB1 and BES1 alters the phosphorylation status of BES1 and increases the ratio of dephosphorylated to phosphorylated BES1, which leads to accumulation of dephosphorylated BES1 in the nucleus. Finally, AGB1 promotes BES1 binding to BR target genes and stimulates the transcriptional activity of BES1. Taken together, our results demonstrate that AGB1 positively regulates cell elongation by affecting the phosphorylation status and transcriptional activity of BES1.

## Introduction

Plants are sessile organisms and their growth and development are controlled by multiple endogenous hormones. Brassinosteroids (BR) are a class of plant steroid hormones that mediate cell elongation, cell division, pathogen resistance, seed germination, stomata formation, flowering, and photomorphogenesis (Azpiroz et al., 1998; Yamamoto et al., 2007; Gao et al., 2008; Gonzalez-Garcia et al., 2011; Oh et al., 2012; Wang et al., 2012; Bekh-Ochir et al., 2013). It has been shown that deficiency in BR biosynthesis or signal transduction causes severe growth defects such as dwarfism, male sterility, delayed senescence, and light-grown phenotype in the dark (Li and Chory, 1997). Specifically, the loss of function mutants of BR, including *de-etiolated 2* (*det2*), *BR-insensitive* (*bin2*), and *dwf4-102* of *Arabidopsis* display dwarfism, short and thick hypocotyls, accumulation of anthocyanins, and open cotyledons (Chory et al., 1991; Li and Chory, 1997; Azpiroz et al., 1998), which are dramatically different from wild-type (WT) phenotypes. Taken together, these phenotypic differences demonstrate that BRs are pivotal regulators of cell elongation.

Unlike animal steroids that primarily bind to nuclear receptors to directly activate target genes (Mangeldorf et al., 1995), BR bind to a cell-surface receptor kinase BR INSENSITIVE1 (BRI1), then initiate a phosphorylation cascade and regulate downstream gene expression by modulating the phosphorylation status and stability of key transcription factors (Vert et al., 2005). Currently, all major components of the BR signaling pathway have been identified in *Arabidopsis*, and the molecular mechanisms of each step of signal transduction also have been studied in detail. When BR bind to the BRI1 receptor, BR signaling kinase (BSK) family protein kinases are phosphorylated (Kinoshita et al., 2005). Then the activated BSK phosphorylates and activates the BRI1 SUPPRESSOR1 (BSU1), a protein phosphatase 1 type phosphatase (Mora-Garcia et al., 2004; Kim et al., 2009, 2011). Subsequently, BSU1 dephosphorylates and inactivates BRASSINOSTEROID INSENSITIVE2 (BIN2), a glycogen synthase kinase 3 (GSK3)-like kinase (Li and Nam, 2002; Kim et al., 2009). As a result, two GSK-substrate transcription factors, BRASSINAZOLE RESISTANT1 (BZR1) and BRI1-EMS-SUPPRESSOR 1 (BES1, also known as BZR2) are dephosphorylated, move into the nucleus, and bind to promoter sequences of BR-responsive genes to enhance BR responses (Kim and Wang, 2010; Sun et al., 2010). As the negative regulator of BR signaling, BIN2 is active when the level of BR is low, and it phosphorylates and inactivates the transcription factors BZR1 and BES1 to suppress BR responses (He et al., 2002). Conversely, when the level of BR is high, BIN2 is inactivated, which causes the protein phosphatase 2A (PP2A) to dephosphorylate BZR1 and BES1 and enhances BR signaling (He et al., 2005; Yin et al., 2005; Sun et al., 2010; Tang et al., 2011).

Guanine nucleotide-binding protein (G-protein) are vital signal transducers composed of three subunits, namely Gα, Gβ, and Gγ, which regulate diverse cellular processes (Jones and Assmann, 2004). Classically, G-protein heterotrimers are activated by G-protein-coupled receptors (GPCRs) that induce the exchange of GDP for GTP at a binding site on Gα (Sprang, 1997). Then the Gα and Gβγ are dissociated from the cytoplasmic face of the plasma membrane and interact with downstream effector proteins (Klein et al., 2000). G-protein signaling is terminated after the Gα hydrolyzes GTP. Mammals have 21 Gα genes, 5 Gβ genes, and 12 Gγ genes, whereas the *Arabidopsis* genome only contains one Gα gene (*GPA1*), one Gβ gene (*AGB1*), and three Gγ genes (*AGG1*–*AGG3*) (Assmann, 2002; Chakravorty et al., 2011; Urano et al., 2013; Urano and Jones, 2014). It has been established that the Gβ plays an essential role not only in the cytoplasm but also in the nucleus. For example, in animals, Gβ interacts with the cytoskeleton to regulate cellular dynamics (Popova and Rasenick, 2003; Layden et al., 2008), and mediates processes in the mitochondria (Zhang et al., 2010), endoplasmic reticulum (Dupre et al., 2006), and Golgi apparatus (Irannejad and Wedegaertner, 2010). Additionally, Gβ of animals also affects the transcriptional activity in the nucleus of proteins including the Fos transcription factor, the muscle differentiation factor myocyte enhancer factor 2 (MEF2) and the adipocyte enhancer-binding protein (AEBP1) (Khan et al., 2013). Similarly, in *Arabidopsis*, AGB1-green fluorescent protein (GFP) fusion or AGB1-mCherry showed that AGB1 can localize to the plasma membrane or the nucleus (Anderson and Botella, 2007; Yu et al., 2016). Most recently, Xu et al. (2017) demonstrated that AGB1 directly interacts with transcription factor BBX21 to inhibit its transcriptional activation activity and promote hypocotyl elongation in *Arabidopsis* (Xu et al., 2017).

It has been shown that mutations in *GPA1*, *AGB1*, and *AGG1-3* alter the sensitivity of plants to multiple hormones, including auxin, ABA, gibberellins, BR, and jasmonic acid (Ullah et al., 2001, 2002, 2003; Wang et al., 2001; Chen et al., 2003), which suggests that G-protein can affect multiple hormone responses to regulate developmental processes in plants. In *Arabidopsis*, a loss-of-function mutation of the Gβ, *agb1*, results in hyposensitivity to BR in seed germination assays (Chen et al., 2004). Furthermore, compared with the WT, BRZ treatment causes rounder leaves and shorter petioles in *agb1* mutants, which is similar to the phenotypes of mutants which have severe defects in BR biosynthesis or BR signaling (Tsugama et al., 2013). Exogenously added BR induces hypocotyl elongation in *agb1*, but the elongation is less than that seen in WT seedlings (Tsugama et al., 2013), which suggests that AGB1 is involved in BR signaling and plays a significant role in BR responses.

Here, we demonstrate that AGB1 interacts with transcription factor BES1 and mediates BR signaling in a BES1-dependent manner. Furthermore, the interaction between AGB1 and BES1 alters the abundance of dephosphorylated and phosphorylated BES1, promoting the transcriptional activity of BES1. Taken together, our results suggested a potential mechanism by which AGB1 modulates BR signaling in a BES1-dependent manner.

## Materials and Methods

### Plant Materials and Growth Conditions

In our study, Columbia ecotype (Col) was used as WT. The *agb1-2* (CS6536) T-DNA line was obtained from the *Arabidopsis* Biological Resource Center (ABRC). *bes1-D* was described previously (Yin et al., 2002). The *bes1-D agb1-2*-double mutant was obtained by crossing the homozygotes of *agb1-2* and *bes1-D*, and the homozygous double mutant *bes1-D agb1-2* was confirmed by PCR and sequencing analysis.

For phenotype analyses, seedlings were grown on 1/2 strength Murashige and Skoog (MS) medium plus 1% (w/v) sucrose with or without the brassinolide (BL) (B1439; Sigma) or BRZ (SML1406; Sigma) (concentrations are shown in the figures). Seedlings for other assays in our study were also sown on the MS medium. These seedlings were incubated for 3 days at 4°C (stratification). Then, the seedlings were placed in white light overnight before being transferred into appropriate light conditions for another 5–6 days at 22°C.

### Plasmid Construction and Transgenic Plants

All constructs were obtained by PCR amplification of the related fragments and cloning into the corresponding vectors. All the constructs and primers used in this study are listed in Supplementary Table S1.

The construct of *35S::BES1-GFP-Flag* and *35S::BES1-Flag* were transformed into *Agrobacterium tumefaciens* (strain GV3101) by the floral dip method (Clough and Bent, 1998), respectively. Transgenic lines were selected on 1/2 MS medium containing 50 mg/ml kanamycin and confirmed by Western blot analysis.

### BL and BRZ Treatments

BL and BRZ stocks were prepared in 80% ethanol and DMSO, respectively. For phenotype analyses, seedlings were grown on 1/2 MS medium plus 1% (w/v) sucrose supplemented with a gradient of BL (0, 1, 10, and 100 nM) or BRZ (0, 0.5, 1, and 2 μM) and then were photographed for measuring hypocotyl length.

For the Western blot analysis of BES1 phosphorylation states, semi-*in vivo* pull down, and quantitative Real-Time PCR assay, 6-day-old light-grown seedlings were incubated in liquid MS medium supplemented with BL or BRZ (concentrations are shown in the figures) under light for the indicated periods of time, and then collected for the subsequent experiments.

### Hypocotyl Measurement

Seedlings were neatly stacked on agar plates. Then seedlings were photographed and the hypocotyl length was measured with Image J software^1^.

### Yeast Two-Hybrid Assay

For the LexA yeast two-hybrid system, combinations containing the fragments fused in pLexA and pB42AD vectors, together with p8op-LacZ vectors, were co-transformed into EGY48 strain by the lithium acetate transformation procedure, as described in the yeast protocols handbook (Clontech Laboratories, Mountain View, CA, United States). Transformed colonies were selected on SD-Trp-Leu-His-Ura (SD-T-L-H-U) medium. The calculation of relative β-galactosidase activity and plate assays were performed as described previously (Yang et al., 2000, 2001; Lian et al., 2011). The GAL4 yeast two-hybrid assay was performed according to the manufacturer’s instructions (Clontech Laboratories, Mountain View, CA, United States). Combinations containing the fragments fused in pGBKT7 and pGADT7 vectors were co-transformed into AH109 strain via the PEG/LiAc transformation procedures. Three days after transformation, yeast cells were spread on the SD-Trp/Leu/His/Ade (SD-T-L-H-A) plates for interaction test. The primer sequences used for plasmid construction for the yeast two-hybrid assay are listed in Supplementary Table S1.

### Pull Down Assay

GST-BES1, His-AGB1, and histidine-tagged trigger factor (His-TF) proteins were expressed in *Escherichia coli* (Rosetta; Kang Wei, Shanghai, China). His-TF was expressed from *E. coli* harboring the empty pCOLD-TF vector, which acted as negative control. The bait protein GST-BES1 was firstly incubated with 20 μl glutathione beads (MagneGST Glutathione Particles; Promega, Madison, WI, United States) in lysis buffer without EDTA at 4°C for 2 h, and washed three times with the same buffer. Then the beads were resuspended in 1 ml lysis buffer without EDTA and the prey proteins His-AGB1 and His-TF were added to the solution. The mixture was incubated for another 2 h at 4°C then washed with lysis buffer without EDTA three times. Proteins were eluted into the 1× SDS loading buffer, boiled for 5 min, and analyzed by Western blot. The prey proteins were detected by anti-His antibody (NewEast, Wuhan, China) and bait proteins were detected by anti-GST antibody (Sigma). The primer sequences used for plasmid construction for the pull-down assay are listed in Supplementary Table S1.

### Semi-*in Vivo* Pull Down Assay

The semi-*in vivo* pull-down assays were performed as described previously (Cai et al., 2014; Yang et al., 2017). GST and GST-AGB1 proteins were expressed in *E. coli* (Rosetta; Kang Wei, Shanghai, China), and were incubated with glutathione Sepharose beads for 2 h at 4°C. *35S::BES1-GFP-Flag* transgenic seedlings were treated with 2 μM BL for 2 h, then ground to powder in liquid nitrogen and solubilized with lysis buffer (50 mM Tris–HCl, pH 7.5, 150 mM NaCl, 1 mM EDTA, 10% glycerol, 0.2% Trition-X-100, 1 mM Pefabloc, 1 mM cocktail, 50 μM MG132). The extracts were centrifuged twice at 13,000 rpm for 10 min and the supernatants were collected and incubated with glutathione Sepharose beads containing GST-AGB1 or GST at 4°C for 2 h. The beads were washed three times with lysis buffer and boiled with 1× SDS loading buffer. The BES1–GFP–Flag proteins pulled down by GST-AGB1 were detected with anti-Flag antibody Flag (Sigma), and bait proteins, GST-AGB1, and GST were visualized by Coomassie Brilliant Blue staining (CBB staining). The primer sequences used for plasmid construction for the semi-*in vivo* pull down assays are listed in Supplementary Table S1.

### Co-immunoprecipitation Assay

*Agrobacterium tumefaciens* (GV3101) containing the *35S::mBES1-GFP-Flag* expression vector was infiltrated alone or together with *A. tumefaciens* (GV3101) harboring *35S::AGB1-Myc-HA* into the leaves of tobacco plants (*Nicotiana benthamiana*). About 48 h after infiltration, the transformed tobacco leaves were collected for the co-immunoprecipitation (Co-IP) assay (Gampala et al., 2007). Samples were homogenized in lysis buffer. After centrifugation, protein supernatant was incubated with 10 μl protein G magnetic beads (Invitrogen), which were previously incubated with 1 μl of anti-Myc antibody (Millipore) overnight at 4°C, for 1 h at 4°C. Then the beads were washed three times with lysis buffer and eluted into 1× SDS loading buffer, boiled for 5 min, and analyzed by Western blot. The mBES1–GFP–Flag pulled down by AGB1–Myc-HA was detected with anti-Flag antibody (Sigma) and AGB1–Myc-HA was detected with anti-HA antibody (Sigma). The primers used for plasmid construction for the Co-IP experiment are listed in Supplementary Table S1.

### Protein Colocalization Assay

*Agrobacterium tumefaciens* (GV3101) harboring indicated constructs or p19 plasmid as diluted in MS medium to OD_600_ = 0.6. Then a mixture of *A. tumefaciens* harboring CFP- and YFP-fusion constructs and p19 plasmid was infiltrated into tobacco leaves at a ratio of 1:1:1 (volume ratio). After 40–48 h, the leaves were collected and analyzed by Confocal Microscopic Examination (Leica TCS SP5II Confocal Laser Scanning Microscope). The primers used for plasmid construction for protein colocalization assay were listed in Supplementary Table S1.

### RNA Extraction and Quantitative Real-Time PCR

Total RNA was extracted according to manufacturer’s instructions (TIANGEN). Detailed method of cDNA synthesis and qRT-PCR was described previously (Zhang et al., 2014; He et al., 2015; Wang et al., 2016). The PP2A was used as an internal control. The primers used are listed in Supplementary Table S2.

### Yeast One-Hybrid Assay

Yeast one-hybrid assay was described previously (Li et al., 2010; Zhang et al., 2017). The pB42 transcriptional activation domain (AD) (45) fused to full-length BES1 and the GAL4 AD fused to full-length AGB1 were cotransformed with the pLacZ-2μ reporter fused to the *DWF4* promoter into the yeast strain EGY48. The transformants were grown on appropriate dropout plates containing X-gal (5-bromo-4-chloro-3-indolyl-β-D-galactopyranoside) for blue color development. Yeast transformation and β-galactosidase activity assay were conducted as described in the Yeast Protocols Handbook (Clontech Laboratories, Mountain View, CA, United States). The primers used for plasmid construction are listed in Supplementary Table S1.

### Dual-LUC Assay

Constructs used in this assay were transformed into *A. tumefaciens* (GV3101). These *A. tumefaciens* cultures were resuspended in MS medium to OD_600_ = 0.6, and then incubated at room temperature for 3 h. The reporter strain harboring *DWF4pro::LUC* was mixed with the effector strains harboring *35S::BES1-Flag* or *35S::cLUC-Flag* (for negative control) and *35S::AGB1-NLS-YFP* or *35S::GUS-NLS-YFP* (for negative control) at the ratio of 2:1:1 (volume ratio). The mixture that lacked any one of these three strains was supplemented with an equal amount of the control MS medium instead. Then the mixture of *A. tumefaciens* suspensions was infiltrated into tobacco leaves. The leaf samples were collected after 3 days under dim light and the dual-luciferase (LUC) assay was carried out using commercial Dual-LUC reaction reagents, according to the manufacturer’s instructions (Promega, Madison, WI, United States). Four biological replicates were measured for each sample. The primers used for Dual-LUC assay are listed in Supplementary Table S1.

### Western Blot Analysis of BES1 Phosphorylation States

Western blot assay was described previously (Sang et al., 2005). The growth conditions and BL and BRZ treatments are described above. Seedlings were collected and homogenized in lysis buffer. The protein was quantified with Bradford assay (Bio-Rad), and subjected to Western blotting analyses with anti-Flag antibody (Sigma–Aldrich) and anti-Actin (Abmart), respectively. The Col and *agb1-2* served as a control. The pBES1 protein level was measured with Image J software^2^.

## Results

### AGB1 Interacts with BES1 in Yeast Cells and *in Vitro*

**FIGURE 1:**
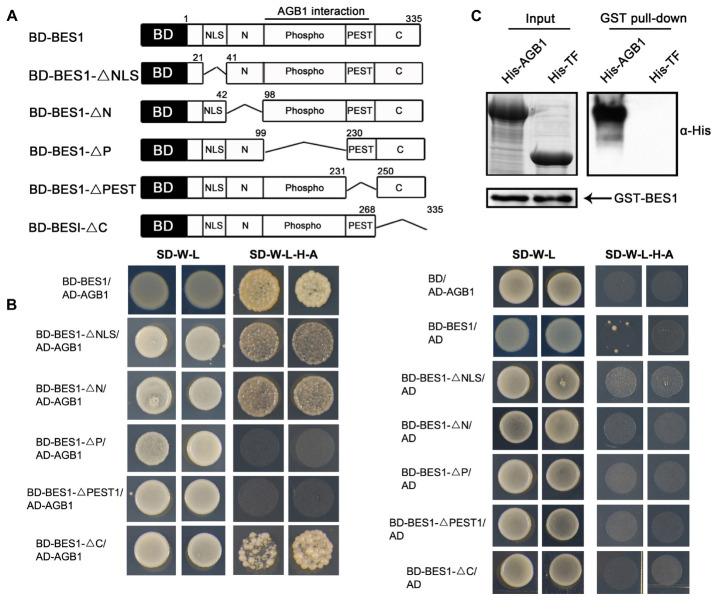
AGB1 interacts with BES1 *in vitro*. **(A)** Schematic structure of various BES1 fragments fused to the GAL4 DNA-binding domain (BD) for yeast two-hybrid assays. **(B)** The P and PEST domain of BES1 are necessary and sufficient for the interaction of AGB1 with BES1. Yeast cells co-expressing the indicated combinations of constructs were grown on basic (SD-T-L) or selective (SD-T-L-H-A) media for 5 days. **(C)** AGB1 interacts with BES1 by pull down assay *in vitro*. GST-BES1 pulls down His-AGB1, but not His-TF (negative control). AGB1 interaction with BES1 was detected with anti-His antibody.

Given previous demonstrations that the G-protein β subunit interacts with transcription factors to regulate their transcriptional activity in mammals, and that AGB1 participates in BR signaling (Tsugama et al., 2013), we asked whether AGB1 interacts directly with the BR-responsive transcription factor BES1. To test this possibility, we first performed yeast two-hybrid assays with bait constructs expressing the GAL4 DNA-binding domain (BD) fused to full-length BES1 and several truncated fragments lacking the NLS (BD-BES1ΔNLS), N (BD-BES1ΔN), P (BD-BES1ΔP), PEST (BD-BES1ΔPEST), and C (BD-BES1ΔC) domains, respectively (**Figure 1A**). Each bait construct was tested for interaction with a prey construct expressing the GAL4 AD fused to full-length AGB1 (AD-AGB1). Growth on selective media was observed for most combinations of bait and prey; however, no growth was observed when BD-BES1ΔP or BD-BES1ΔPEST was used as bait with AD-AGB1 prey (**Figure 1B**). These results indicated that AGB1 directly interacts with BES1 in yeast cells and that the P and PEST domains are indispensable for the interaction between AGB1 with BES1.

We confirmed the interaction of between AGB1 and BES1 by performing a pull down assay. Fragments encoding the full-length BES1 and AGB1 were cloned into pGEX-4T-1 and pCOLD-TF vectors, and the GST-tagged BES1 and His-tagged AGB1 fusion proteins were expressed and purified from *E. coli.* His-TF was expressed and purified from *E. coli* harboring the empty pCOLD-TF vector, which served as negative control. Consistent with the yeast two-hybrid results, AGB1 was strongly pulled down by BES1 (**Figure 1C**). Thus, AGB1 interacts with BES1 directly *in vitro*.

### AGB1 Physically Interacts with BES1 in the Plant Cell

**FIGURE 2:**
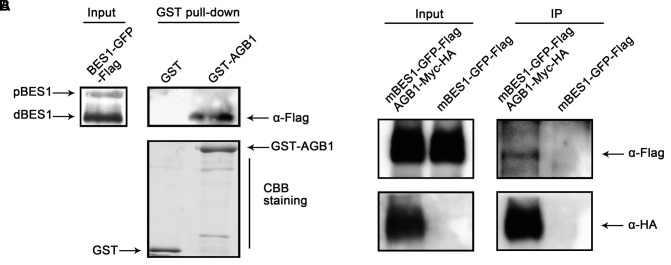
AGB1 interacts with BES1 *in vivo*. **(A)** In a semi-*in vivo* pull down assay, AGB1 was found to interact with dephosphorylaged BES1. GST and GST-AGB1 proteins expressed from *E. coli* were used to pull down BES1 from whole protein extracts of *35::BES1-GFP-Flag Arabidopsis* seedlings. The presence of phosphorylated (pBES1) or dephosphorylaged (dBES1) BES1–GFP–Flag protein was detected by Western blotting with anti-Flag antibodies. GST and GST-AGB1 were detected by Coomassie Brilliant Blue staining (CBB staining). **(B)** AGB1 interacts with BES1 by co-immunoprecipitation assay in tobacco cells. Total protein extracted from tobacco leaves co-expressing the mBES1–GFP–Flag and AGB1–Myc-HA or mBES1–GFP–Flag alone were immunoprecipitated (IP) by Myc antibody-conjugated protein-G magnetic beads. mBES1–GFP–Flag was detected by anti-Flag antibodies and AGB1–Myc-HA was detected by anti-HA antibodies.

The interaction between AGB1 and BES1 *in vitro* prompted us to explore whether AGB1 physically interacts with BES1 in plant cells. We first tested this using a semi-*in vivo* pull down assay, and found that AGB1 pulls down BES1 from extracts of *Arabidopsis* seedlings treated with BL (**Figure 2A**). To further confirm the interaction between AGB1 and BES1 *in vivo*, we performed a Co-IP assay with tobacco leaves co-expressing Flag and GFP-tagged BES1 and Myc and HA-tagged AGB1 proteins. However, we could not detect the expression of the BES1–GFP–Flag proteins; therefore, we used the mBES1–GFP–Flag, which harbors a mutation in the PEST motif (Wang et al., 2002; Yin et al., 2002, 2005). Compared with BES1 proteins, the mBES1 proteins accumulate higher levels of both phosphorylated and dephosphorylated BES1 (Vert et al., 2005). Our results indicated that mBES1–GFP–Flag is co-precipitated by AGB1–Myc-HA (**Figure 2B**). In conclusion, AGB1 interacts with BES1 *in vivo*.

### AGB1 Is Involved in BR Response

**FIGURE 3:**
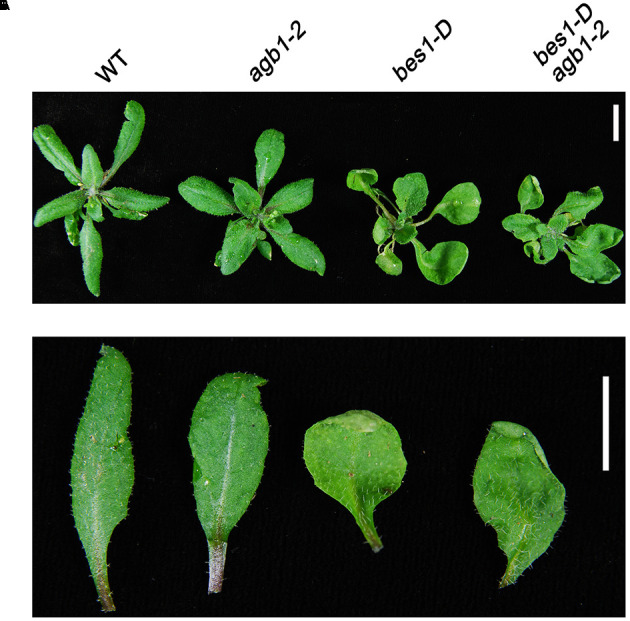
AGB1 is involved in the BR signaling pathway. Phenotypes of rosettes **(A)** and detached leaves **(B)** wild-type, *agb1-2*, *bes1-D*, and *bes1-D agb1-2.* The *agb1-2* mutation partially alleviates the effect of *bes1-D* on leaf morphology. Seedlings were grown on aaa strength MS medium containing 1% w/v sucrose for 2 weeks and then were transplanted to soil and grown for 3 weeks under continuous white light. Scale bar = 1 cm.

To explore how the interaction between BES1 and AGB1 affects plant growth, we crossed *agb1-2*, a null mutant of AGB1, with *bes1-D*, a gain-of-function mutant of BES1 exhibiting constitutive BR-response phenotypes (Yin et al., 2002). The *bes1-D* and *agb1-2* mutants display different leaf morphologies. *bes1-D* displays long and bending petioles and curly leaves, whereas the leaves and petioles of the *agb1-2* do not show these phenotypes (**Figure 3A**). Interestingly, compared with the leaf phenotype of *bes1-D*, the double mutant *bes1-D agb1-2* displays shorter petioles and slightly curly leaves, which suggested that the mutation of AGB1, *agb1-2*, alleviates the constitutive activity of *bes1-D* and reduces BR responses (**Figures 3A,B**). These differences in leaf morphology indicate that the AGB1 plays a role in the regulation of BR responses.

### AGB1 Is a Positive Regulator in the BR Pathway

**FIGURE 4:**
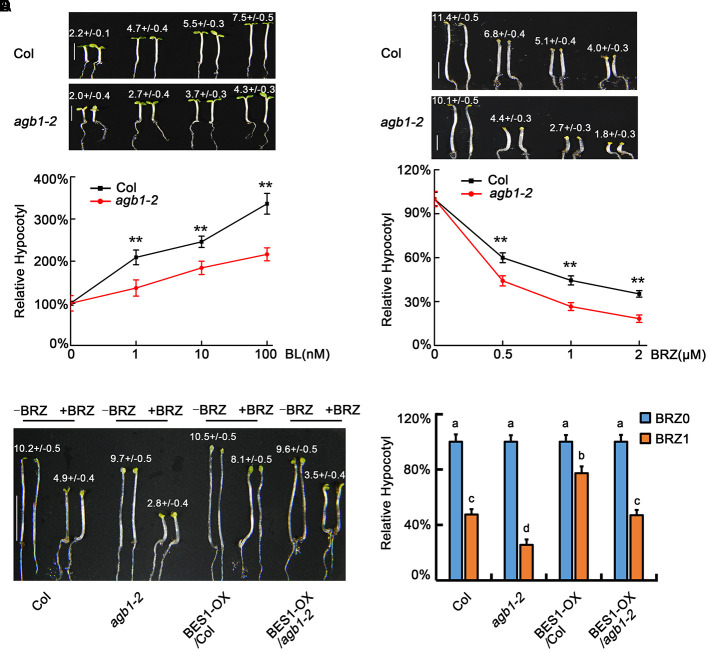
AGB1 positively regulates BR signaling pathway. **(A,B)** Photographs and relative hypocotyl lengths of Col and *agb1-2* in the presence of 0, 1, 10, or 100 nM BL. Seedlings were grown under 8–10 μmol/m^2^/s blue light for 5 days. Scale bar = 2.5 mm. Values are means ± SD. Values in **(A)** are the absolute hypocotyl lengths of seedlings. Asterisks in **(B)** denote significant difference between the indicated samples (Student’s *t*-test, *P* ≤ 0.01, compared to Col). Error bars in **(B)** represent means ± SD (*n* > 25) and the hypocotyl lengths of seedlings in the absence of BL were defined as “100%.” **(C,D)** Photographs and relative hypocotyl lengths of Col and *agb1-2* in the presence of 0, 0.5, 1, or 2 μM BRZ. Seedlings were grown in the darkness for 5 days. Scale bar = 2.5 mm. Values are means ± SD. Values in **(C)** are the absolute hypocotyl lengths of seedlings. Asterisks in **(D)** denote significant difference between the indicated samples (Student’s *t*-test, *P* ≤ 0.01, compared to Col). Error bars in **(D)** represent means ± SD (*n* > 25) and the hypocotyl lengths of seedlings in the absence of BRZ were defined as “100%.” **(E,F)** Photographs and relative hypocotyl lengths of Col, *agb1-2*, BES1-Flag/Col, and BES1-Flag/*agb1-2* in the absence or presence of 1 μM BRZ. Seedlings were grown in the darkness for 5 days. Scale bar = 5 mm. Values are means ± SD. Values in **(E)** are the absolute hypocotyl lengths of seedlings. The letters “a”–“d” denote statistically significant differences between the indicated samples, as determined by Tukey’s LSD test (*P* ≤ 0.01). Error bars in **(F)** represent means ± SD (*n* > 25) and the hypocotyl lengths of seedlings in the absence of BRZ were defined as “100%.”

To further identify the physiological roles of AGB1, we tested the sensitivity of *agb1-2* and WT to BL and BRZ. A previous study showed that the hypocotyls of *agb1-2* are shorter than the WT when grown in the dark or under light (Chen et al., 2004). Furthermore, our results showed that the elongation of hypocotyl in *agb1-2* is less than in WT seedlings exposed to the same concentrations of BL (**Figures 4A,B**). In contrast to BL treatment, BRZ inhibits hypocotyl elongation, and this inhibition was greater in *agb1-2* than in the WT (**Figures 4C,D**). In summary, *agb1-2* is hyposensitive to BR and hypersensitive to BRZ, which implies that AGB1 is a positive regulator in BR response pathway.

To further analyze the role of AGB1 is BR signaling, we overexpressed BES1 in the Col and *agb1-2* backgrounds to produce *35S::BES1-Flag/*Col and *35S::BES1-Flag/agb1-2* transgenic lines. Analysis of the hypocotyl phenotype showed that the hypocotyls of *35S::BES1-Flag/agb1-2* were significantly taller than those of *agb1-2* (**Figures 4E,F** and Supplementary Figure S1), which indicated the overexpression of BES1 alleviates the response to BRZ in *agb1-2*. Considering that both BZR1 and BES1 are the key transcription factors of BR signaling, these results suggest that AGB1 regulation of hypocotyl elongation proceeds, at least in part, through regulation of BES1.

### AGB1 Positively Modulates the Expression of BR-Responsive Genes

**FIGURE 5:**
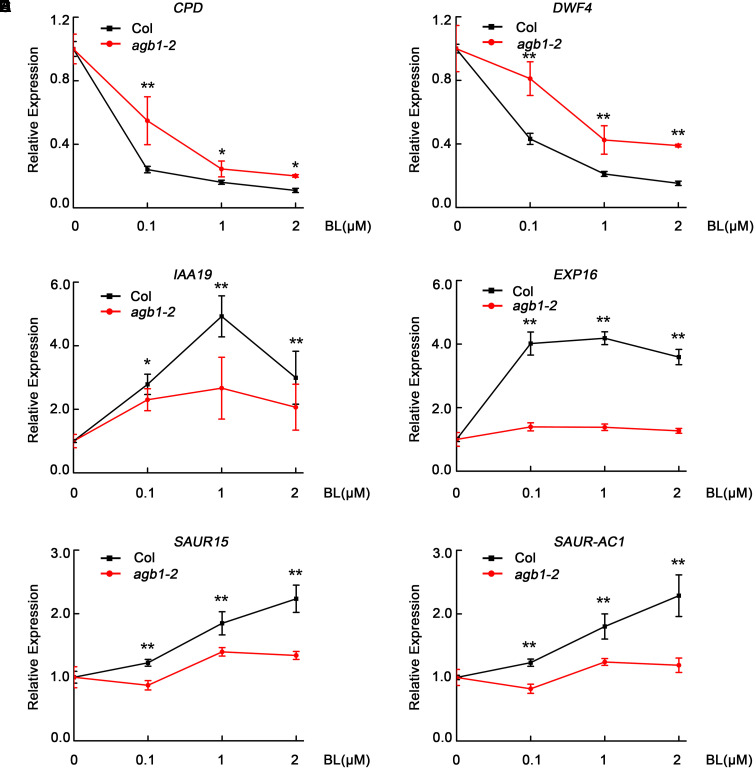
AGB1 modulates the expression of BR-responsive genes. **(A–F)** qRT-PCR analysis of *CPD*, *DWF4*, *IAA19*, *EXP16*, *SAUR15*, and *SAUR-AC1* in Col and *agb1-2.* Seedlings were grown in white light for 5 days and then were treated with 0, 0.1, 1, or 2 μM BL for 2 h in light. The expression of each target gene in seedlings treated with 0 μM BL was defined as “1.” Error bars represent means ± SD (*n* = 3). One asterisk denotes significant difference between the indicated samples (Student’s *t*-test, *P* ≤ 0.05, compared to Col). Two asterisks denote significant difference between the indicated samples (Student’s *t*-test, *P* ≤ 0.01, compared to Col).Values were normalized to the expression of PP2A.

With the results that AGB1 interacts with BES1 and enhances the BR response, we hypothesized that AGB1 and BES1 may co-regulate the expression of BR-responsive target genes. To test this, we performed quantitative RT-PCR analysis of six typical BR-responsive genes, *CPD*, *DWF4*, *IAA19*, *EXP16*, *SAUR15*, and *SAUR-AC1*. It has been reported that *CPD* and *DWF4* are BR-repressed genes and the other are BR-activated genes, which act to promote hypocotyl elongation (Oh et al., 2012; Kim et al., 2014; Jiang et al., 2015). In this assay, WT Col and *agb1-2* seedlings were grown in white light for 5 days and then were subjected to BL treatment (at 0, 0.1, 1, or 2 μM) for 2 h. We found that expression of the BR-repressed genes *CPD* and *DWF4* decreased less in *agb1-2* than in Col, with expression decreasing more in both genotypes as BL concentration increased (**Figures 5A,B**). By contrast, higher expression of the BL-induced genes *IAA19*, *EXP16*, *SAUR15*, and *SAUR-AC1* was detected in the Col than in *agb1-2* (**Figures 5C–F**). These results are consistent with the finding that *agb1-2* is hyposensitive to BR and support the conclusion that AGB1 cooperates with BES1 to regulate the BR-responsive target genes.

### AGB1 Activates BR Signaling by Reducing the Abundance of Phosphorylated BES1

**FIGURE 6:**
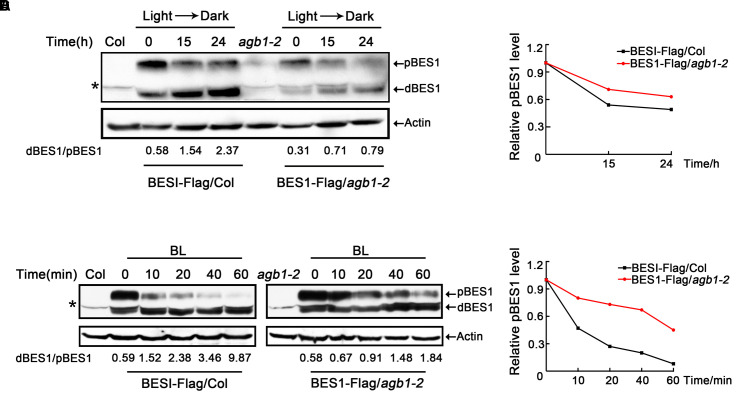
AGB1 affects the phosphorylation status of BES1. **(A,C)** Western blot analysis of phosphorylation status of BES1 in Col or *agb1-2* plants expressing BES1–Flag in darkness **(A)** and under BL treatment **(B)**. Six-day-old BES1–Flag transgenic seedlings were kept in darkness or treated with BL for the indicated periods of time and used to prepare protein to detect the dephosphorylated BES1 (dBES1) and phosphorylated BES1 (pBES1) with anti-Flag antibody. Actin was used as a loading control. Data indicate the ratio of dBES1/pBES1. The upper arrow denotes pBES1, the lower arrow denotes dBES1. Asterisk denotes non-specific band. **(B,D)** Analysis of pBES1 protein level in Col or *agb1-2* plants expressing BES1–Flag in darkness **(A)** and under BL treatment **(C)**. The pBES1 protein level in the absence of darkness **(A)** and BL **(C)** was defined as “1.0.”

Previous genetic screens have identified that BES1 is a positive regulator of the BR signaling pathway and exists as two different forms, namely the phosphorylated form (pBES1) and the dephosphorylated form (dBES1). dBES1 can accumulate on the nucleus, which is critical for BR signaling (He et al., 2002; Yin et al., 2002). Moreover, fusion of BZR1 and BES1 to fluorescent proteins has shown that the dephosphorylated forms of both proteins accumulate in the nucleus following BL treatment, rather than the phosphorylated forms (He et al., 2002; Wang et al., 2002; Yin et al., 2002), which suggests that dBES1 is the active form and more important in regulating BR signaling and plant development. Furthermore, Kim et al. (2014) found that darkness increases the activity of the BR-specific transcription factor, BZR1, by decreasing the phosphorylated form of BZR1 in a proteasome-dependent manner (Kim et al., 2014). Based on these studies, we examined whether AGB1 can affect the phosphorylation status of BES1, the close homolog of BZR1, to regulate the BR signaling pathway. To do this, we investigated the abundance of the pBES1 and dBES1 in seedlings grown in darkness. Transgenic seedlings expressing *35S::BES1-Flag/*Col and *35S::BES1-Flag/agb1-2* were grown in light for 5 days, then were transferred to darkness for 0, 15, or 24 h, respectively. The ratio of dBES1 to pBES1 (dBES1/pBES1) was used to determine the status of dephosphorylated BES1, as in the previous study (Kim et al., 2014). Time course analysis of *35S::BES1-Flag/*Col showed that the dBES1/pBES1 increases rapidly; however, the dBES1/pBES1 of *35S::BES1-Flag/agb1-2* increases more slowly (**Figure 6A** and Supplementary Figure S4A). These results suggest that AGB1 may enhance BR signaling by increasing the ratio of dBES1/pBES1.

We also tested whether AGB1 regulates the phosphorylation state of BES1 in response to BL treatment. After subjecting *35S::BES1-Flag/*Col, and *35S::BES1-Flag/agb1-2* seedlings to 2 μM BR for 0, 10, 20, 40, or 60 min, we found that the dBES1/pBES1 increases in both genetic backgrounds (**Figure 6C** and Supplementary Figure S4C), which is consistent with the previous study (Kim et al., 2014). Interestingly, compared to *35S::BES1-Flag/agb1-2*, the increase of dBES1/pBES1 in *35S::BES1-Flag/*Col is dramatically higher (**Figure 6C** and Supplementary Figure S4C), which indicates that the mutation in AGB1 inhibits the response to BL. Moreover, we also analyzed the pBES1 protein level, and the results showed that pBES1 are degraded much faster in *35S::BES1-Flag*/Col than in *35S::BES1-Flag*/*agb1-2* both in darkness and BL treatment (**Figures 6B,D** and Supplementary Figures S4B,D). Taken together, we confirmed that AGB1 increases the abundance of BES1 in its active, dephosphorylated state by promoting the degradation of phosphorylated BES1.

### AGB1 Cooperates with BES1 to Promote the Transcriptional Activity of BES1

**FIGURE 7:**
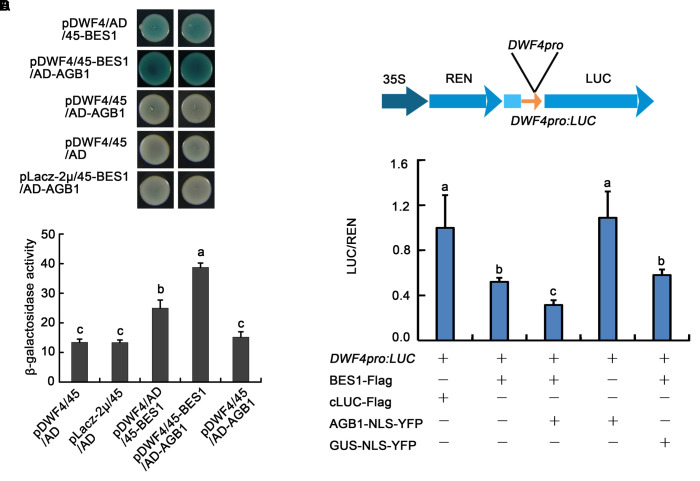
AGB1 regulates the transcription level of BES1 direct-target genes *in vitro* and *in vivo*. **(A)** Yeast one-hybrid assay showing AGB1 promotes BES1 binding to the *DWF4* promoter. Yeast cells were cotransformed with the *DWF4* promoter fused to the *LacZ* reporter gene, in addition to BES1 and/or AGB1 fused to either the GAL4 AD or the pB42 AD (45). Blue color indicates expression of the *LacZ* reporter. When AGB1 is present, BES1 shows stronger transcriptional activity. **(B)** Quantification of β-galactosidase activity for the yeast one-hybrid assay in **(A)**. The letters “a”–“c” denote statistically significant differences between the indicated samples, as determined by Tukey’s LSD test (*P* ≤ 0.01). Error bars represent means ± SD (*n* = 3). **(C)** Schematic representation of the dual-LUC assay reporter construct expressing LUC under the *DWF4* promoter (*DWF4pro*). **(D)** Tobacco leaves were infiltrated with strains harboring the *DWF4pro: LUC* reporter and effectors in the indicated combinations. cLUC–Flag and GUS–NLS–YFP served as negative controls for BES–Flag and AGB1–NLS–YFP, respectively. Expression values are determined by calculating the ratio of LUC activity to REN activity (LUC/REN). The letters “a”–“c” denote statistically significant differences between the indicated samples, as determined by Tukey’s LSD test (*P* ≤ 0.05). Error bars represent means ± SD (*n* = 3).

AGB1 mediates the activity of BR signaling pathway through affecting the abundance of dephosphorylated and phosphorylated BES1. To further investigate the relationship between AGB1 and BES1, we performed yeast-one hybrid assays to test whether AGB1 regulates the transcriptional activity of BES1. The results indicate that BES1 binds to the *DWF4* promoter region (**Figure 7A**, Line 1), but AGB1 alone shows no binding activity in our yeast one-hybrid assay (**Figure 7A**, Line 3). Interestingly, when AGB1 and BES1 are co-expressed with the promoter of *DWF4*, BES1 shows stronger transcriptional activity in yeast cells (**Figure 7A**, Line 2). Additionally, we detected the β-galactosidase activity in the yeast cells, which quantifies the expression of the *LacZ* reporter gene, and the results also confirmed that AGB1 enhances the binding activity of BES1 (**Figure 7B**).

We also used an *in vivo* transient transcription assay, the dual-LUC assay, to test whether AGB1 could regulate the transcriptional activity of BES1 in tobacco leaves. The reporter and effectors were transiently co-expressed in tobacco leaves as indicated (**Figure 7D** and Supplementary Figure S5). BES1-Flag could significantly reduce expression of *DWF4pro::LUC*, while cLUC–Flag (negative control) and AGB1–NLS–YFP alone show no effect. When AGB1–NLS–YFP and BES1–Flag are co-expressed in the tobacco leaves, the expression of the reporter *DWF4pro::LUC* is reduced significantly compared with GUS–NLS–YFP (negative control) and BES1–Flag alone (**Figure 7D** and Supplementary Figure S5). This supports our earlier result that AGB1 enhances the transcriptional activity of BES1 to regulate BR response genes.

## Discussion

### The Interaction of AGB1 and BES1 Is the Point of Crosstalk between G-Protein and BR Signaling Pathway

A key question in plant biology is how plants are able to sense and integrate a wide variety of environmental and internal signals to produce the proper growth and developmental responses. Phytohormones, such as auxins, gibberellic acids, abscisic acid, cytokinins, ethylene, BRs, jasmonic acid, and strigolactones, each have their own distinctive functions. However, they also communicate with each other and with other factors including light, sugar, and G-protein, to regulate plant growth in a precise manner (Santner and Estelle, 2009). However, much still remains to be understood about how these integrations between different hormones and signals coordinate plant growth.

Previous studies indicated that the BES1 and BZR1 transcription factors play an essential role in BR signaling (Sun et al., 2010). Additionally, other transcription factors including BIM1, MYB30, and PIF4, chromatin-modifying enzymes REF6 and ELF6, and the transcription elongation factor IWS1 all have been found to interact with BES1 or BZR1 to control plant development (Guo et al., 2013). Here, we identified a key point of crosstalk between G-protein and the BR signaling pathway. This connection is mediated by direct interaction between the G-protein β subunit AGB1 with BES1. First, we demonstrated that AGB1 interacts with BES1 *in vitro*, through yeast two-hybrid and GST pull down assays (**Figures 1A–C**). Moreover, semi-*in vivo* pull down and Co-IP assays revealed the interaction of AGB1 and BES1 in plant cells (**Figures 2A,B**). Further, the physiological phenotype analysis indicated that the AGB1 null mutant *agb1-2* is hyposensitive to BR, but hypersensitive to BRZ, compared with the WT (**Figures 4A–D**). Moreover, the mutation in AGB1 enhances the effect of BRZ in the overexpression of BES1 (Supplementary Figure S1). All of these data suggest that AGB1 positively modulates BR signaling pathway to promote hypocotyl elongation in *Arabidopsis.* Finally, quantitative RT-PCR analysis, Western blot analysis, yeast one-hybrid, and Dual-LUC assays indicated that AGB1 cooperates with BES1 to promote BES1 binding at the promoters of BR-response genes and regulation of their transcription (**Figures 5–7**). This suggests that the interaction between AGB1 and BES1 modulates the crosstalk between BR and G-protein signaling through regulation of transcriptional networks.

A previous yeast two-hybrid assay using BES1 as a bait identified BIM1, a new class of basic helix-loop-helix transcription factor, and demonstrated BES1 and BIM1 are able to form a heterodimer and bind CANNTG E-box motifs in the promoter of BR-responsive genes to mediate BR signaling in a cooperative manner (Yin et al., 2005). We show by LexA yeast two-hybrid assay that full-length AGB1 interacts with BIM1, as demonstrated by high β-galactosidase activity (Supplementary Figures S2A,B). Additionally, AGB1 and BIM1 are localized to the same nuclear bodies via colocalization study in tobacco cells (Supplementary Figure S2C). Taken together, these results demonstrate that AGB1 not only interacts with BES1, but also with BIM1, indicating an even greater role of AGB1 in BR signaling.

### AGB1 Activates BR Signaling by Changing the Phosphorylation State of BES1 Protein

It has been established that the dephosphorylated forms of BZR1 and BES1 and their concomitant accumulation in the nucleus control the activity of BR signaling pathway (Ryu et al., 2007), while their phosphorylated forms remain inactive and unstable and can be degraded by the proteasome under normal conditions (Vert et al., 2005). Moreover, darkness and BL treatment can both promote a shift from pBES1 to dBES1 and result in a strong BR response (He et al., 2002; Wang et al., 2013). Based on these facts, we found that BES1 overexpression plants of different genetic backgrounds, *35S::BES1-Flag/*Col and *35S::BES1-Flag/agb1-2*, show significantly different responses to darkness or 2 μM BL treatment (**Figure 6** and Supplementary Figure S4). We observed that the increase of the dBES1/pBES1 following darkness or BL treatment in *35S::BES1-Flag/*Col highly exceeds that of *35S::BES1-Flag/agb1-2*, which suggests that AGB1 acts as a positive regulator of BR signaling by altering the abundance of dBES1/pBES1.

The proteasome inhibitor MG132 increases the accumulation of phosphorylated BZR1, indicating that its phosphorylated form can be degraded by the proteasome (He et al., 2002). Our yeast two-hybrid assay showed that the deletion of the P and PEST domains, which mediate the phosphorylation status of BES1 and its proteolytic degradation (He et al., 2002), abolishes the ability of BES1 to interact with AGB1 (**Figure 1B**). This suggests that AGB1 may act to influence the phosphorylation status of BES1. Further, Western blot analysis after BL treatment also indicated that the regulation of AGB1 can increase the dBES1/pBES1 ratio by reducing the abundance of pBES1 (**Figure 6** and Supplementary Figure S4). Based on these findings, we propose a mechanism for the function of AGB1 proteins in the BR signaling pathway. The interaction of AGB1 and BES1 likely enhances the BR response by promoting the degradation of the pBES1 and increasing the ratio of dBES1/pBES1.

However, a previous study concluded that AGB1 regulates the BR signaling pathway, but independently of BZR1 and without any effect on BZR1 phosphorylation state (Tsugama et al., 2013). As BZR1 and BES1 have high similarity to each other and perform similar functions in the BR signaling pathway, our results appear to be different. This discrepancy is likely due to the difference in time period for which seedlings were exposed to BL. He et al. (2002) analyzed the effect of BL treatment on BZR1–CFP proteins in transgenic plants by immunoblotting and found that the increase in the BZR1–CFP protein level became obvious after 30 min of BL treatment, but the signal level peaked after 1 h (He et al., 2002), which suggests that longer treatment with BL might eliminate differences in dephosphorylated or phosphorylated BZR1. Thus, longer treatment with BL might be unsuitable for analysis of abundance of dephosphorylated or phosphorylated BZR1. To test whether time of treatment with BL is critical for detection of differences in BES1 phosphorylation state, we assayed levels of BES1 after 15 days of BL treatment, and found no difference between Col and *agb1-2* plants (Supplementary Figure S3). This is the different result of our short-term treatment with BL, in which we detected dramatically different abundance of dBES1/pBES1 between *35S::BES1-Flag/*Col and *35S::BES1-Flag/agb1-2* (**Figure 6C** and Supplementary Figure S4C). It may be that when seedlings are exposed to BL for a long period of time, they adapt to BL and their response to it becomes restrained, which eliminates the effect of the *agb1-2* mutation on BES1 (and potentially BZR1) phosphorylation state. This could explain the difference between our results and those of Tsugama et al. (2013).

### The Interaction of AGB1 and BR Signaling Regulates Hypocotyl Elongation in *Arabidopsis*

As in animals, plant G-protein signaling mediates a wide array of signaling processes. However, unlike animals, plants possess very few G-protein subunit isoforms. One of the central questions about plant G-protein signaling is how such a small set of G-protein isoforms is able to function in multiple developmental and physiological signaling processes. The *Arabidopsi*s and rice G-protein β subunits, AGB1 and RGB1, have been shown to be localized to the plasma membrane, cytosol, and nucleus (Anderson and Botella, 2007). This localization to various cellular compartments may allow for their association with different effectors, and contribute to the ability of G-protein subunits to function in multiple signaling roles. Most recently, Xu et al. (2017) revealed that AGB1 participates in the light response, acting as a molecular switch by binding to the light-activated transcription factor BBX21, which promotes hypocotyl elongation through inhibiting the transcription activation of BBX21 (Xu et al., 2017). In this study, we describe a similar mechanism in which AGB1 interacts physically with the BR hormone-responsive transcription factor BES1 to regulate hypocotyl elongation. Previous studies indicated that *gpa1* can specifically enhance the cell division defects of *bril1-5* and *det2-1* mutants in *Arabidopsis* (Gao et al., 2008). These results indicated that G-protein modulate BR-mediated cell division and cell elongation by different subunits.

**FIGURE 8:**
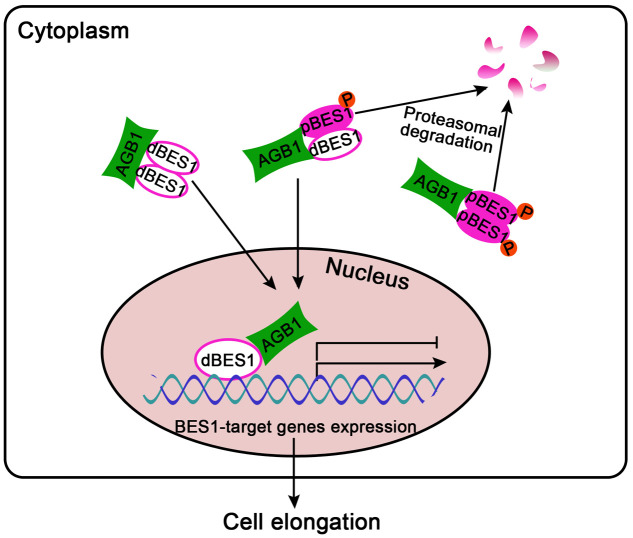
A proposed model for G-protein and BR signaling crosstalk mediated by AGB1 and BES1 interaction. In the cytoplasm, AGB1 interacts with the dimers of pBES1–pBES1, dBES1–dBES1, and pBES1–dBES1, and promotes degradation of pBES1 in a proteasome-dependent manner, leading to a high dBES1/pBES1. Then, the active dBES1 accumulates in the nucleus and regulates downstream gene expression to promote cell elongation. Arrows and bars represent the actions of promotion and inhibition, respectively.

Our results reveal that AGB1 and BES1 link G-protein and BR signaling to regulate hypocotyl elongation. The protein level of BES1 as well as the ratio of dBES1/pBES1 is critical for plant growth (Yang et al., 2017). Our Western blot analysis demonstrated that AGB1 can stabilize dBES1 and increase the dBES1/pBES1 ratio by mediating pBES1 degradation (**Figure 6** and Supplementary Figure S4). Furthermore, we found that AGB1 promotes BES1 binding to the BR target genes and enhances transcriptional activation by BES1 (**Figure 7** and Supplementary Figure S5). Based on these facts, we propose a potential model to illustrate how G-protein and BR signaling pathway cooperate to mediate cell elongation (**Figure 8**). As previously reported, BES1 proteins exist as dimers in pBES1–pBES1, dBES1–dBES1, and pBES1–dBES1 pairs in the cell (Kim et al., 2014). Under the normal condition, pBES1 can trap dBES1 to form a dimer for a greater affinity, and pBES1–pBES1 and dBES1–dBES1 also possibly exist (Kim et al., 2014). In the cytoplasm, AGB1 might interact with the different phosphorylated forms of BES1 and facilitate degradation of pBES1, leading to a high dephosphorylation level of BES1 and the high dBES1/pBES1. In addition, the dBES1 accumulates in the nucleus and AGB1 promotes dBES1 to bind to the promoter of BES1 target genes and stimulates the transcription activity of those genes, controlling cell elongation. The mechanism may accurately and sensitively function to promote the dynamic transition between pBES1 and dBES1 so that seedlings may respond precisely to BR during growth and development. Therefore, the co-regulation of AGB1 and BES1 to G-protein and BR signaling pathway may be key for balancing plant growth and survival.

## Author Contributions

HL, PX, and TZ conceived and designed the work. TZ and PX carried out the experiments. WW and SW helped with constructing some clones. HL, TZ, and PX analyzed the data. TZ and HL wrote the article. PX, JC, and H-QY revised the article.

## Conflict of Interest Statement

The authors declare that the research was conducted in the absence of any commercial or financial relationships that could be construed as a potential conflict of interest.

## Footnotes

1 http://rsbweb.nih.gov/ij/

2 http://rsbweb.nih.gov/ij/

## References

[B1] Anderson D. J. Botella J. R. (2007). Expression analysis and subcellular localization of the *Arabidopsis thaliana* G-protein beta-subunit AGB1. *Plant Cell Rep.* 26 1469–1480. 10.1007/s00299-007-0356-1 17492287

[B2] Assmann S. M. (2002). Heterotrimeric and unconventional GTP binding proteins in plant cell signaling. *Plant Cell* 14 S355–S373. 10.1105/tpc.001792 12045288 PMC151266

[B3] Azpiroz R. Wu Y. Locascio J. C. Feldmann K. A. (1998). An Arabidopsis brassinosteroid-dependent mutant is blocked in cell elongation. *Plant Cell* 10 219–230. 10.1105/tpc.10.2.219 9490745 PMC143978

[B4] Bekh-Ochir D. Shimada S. Yamagami A. Kanda S. Ogawa K. Nakazawa M. (2013). A novel mitochondrial DnaJ/Hsp40 family protein BIL2 promotes plant growth and resistance against environmental stress in brassinosteroid signaling. *Planta* 237 1509–1525. 10.1007/s00425-013-1859-3 23494613 PMC3664749

[B5] Cai Z. Liu J. Wang H. Yang C. Chen Y. Li Y. (2014). GSK3-like kinases positively modulate abscisic acid signaling through phosphorylating subgroup III SnRK2s in *Arabidopsis*. *Proc. Natl. Acad. Sci. U.S.A.* 111 9651–9656. 10.1073/pnas.1316717111 24928519 PMC4084465

[B6] Chakravorty D. Trusov Y. Zhang W. Acharya B. R. Sheahan M. B. Mccurdy D. W. (2011). An atypical heterotrimeric G-protein γ-subunit is involved in guard cell K^+^-channel regulation and morphological development in *Arabidopsis thaliana*. *Plant J.* 67 840–851. 10.1111/j.1365-313X.2011.04638.x 21575088

[B7] Chen J. G. Pandey S. Huang J. Alonso J. M. Ecker J. R. Assmann S. M. (2004). GCR1 can act independently of heterotrimeric G-protein in response to brassinosteroids and gibberellins in Arabidopsis seed germination. *Plant Physiol.* 135 907–915. 10.1104/pp.104.038992 15181210 PMC514125

[B8] Chen J. G. Willard F. S. Huang J. Liang J. Chasse S. A. Jones A. M. (2003). A seven-transmembrane RGS protein that modulates plant cell proliferation. *Science* 301 1728–1731. 10.1126/science.1087790 14500984

[B9] Chory J. Nagpa P. Petob C. A. (1991). Phenotypic and genetic analysis of det2, a new mutant that affects light-regulated seedling development in Arabidopsis. *Plant Cell* 3 445–459. 10.1105/tpc.3.5.445 12324600 PMC160013

[B10] Clough S. J. Bent A. F. (1998). Floral dip: a simplified method for *Agrobacterium*-mediated transformation of *Arabidopsis thaliana*. *Plant J.* 16 735–743. 10.1046/j.1365-313x.1998.00343.x 10069079

[B11] Dupre D. J. Robitaille M. Ethier N. Villeneuve L. R. Mamarbachi A. M. Hebert T. E. (2006). Seven transmembrane receptor core signaling complexes are assembled prior to plasma membrane trafficking. *J. Biol. Chem.* 281 34561–34573. 10.1074/jbc.M605012200 16959776

[B12] Gampala S. S. Kim T. W. He J. X. Tang W. Deng Z. Bai M. Y. (2007). An essential role for 14-3-3 proteins in brassinosteroid signal transduction in Arabidopsis. *Dev. Cell* 13 177–189. 10.1016/j.devcel.2007.06.009 17681130 PMC2000337

[B13] Gao Y. Wang S. Asami T. Chen J. G. (2008). Loss-of-function mutations in the Arabidopsis heterotrimeric G-protein alpha subunit enhance the developmental defects of brassinosteroid signaling and biosynthesis mutants. *Plant Cell Physiol.* 49 1013–1024. 10.1093/pcp/pcn078 18499742

[B14] Gonzalez-Garcia M. P. Vilarrasa-Blasi J. Zhiponova M. Divol F. Mora-Garcia S. Russinova E. (2011). Brassinosteroids control meristem size by promoting cell cycle progression in *Arabidopsis* roots. *Development* 138 849–859. 10.1242/dev.057331 21270057

[B15] Guo H. Li L. Aluru M. Aluru S. Yin Y. (2013). Mechanisms and networks for brassinosteroid regulated gene expression. *Curr. Opin. Plant Biol.* 16 545–553. 10.1016/j.pbi.2013.08.002 23993372

[B16] He J. X. Gendron J. M. Sun Y. Gampala S. S. Gendron N. Sun C. Q. (2005). BZR1 is a transcriptional repressor with dual roles in brassinosteroid homeostasis and growth responses. *Science* 307 1634–1638. 10.1126/science.1107580 15681342 PMC2925132

[B17] He J. X. Gendron J. M. Yang Y. Li J. Wang Z. Y. (2002). The GSK3-like kinase BIN2 phosphorylates and destabilizes BZR1, a positive regulator of the brassinosteroid signaling pathway in *Arabidopsis*. *Proc. Natl. Acad. Sci. U.S.A.* 99 10185–10190. 10.1073/pnas.152342599 12114546 PMC126645

[B18] He S. B. Wang W. X. Zhang J. Y. Xu F. Lian H. L. Li L. (2015). The CNT1 domain of Arabidopsis CRY1 alone is sufficient to mediate blue light inhibition of hypocotyl elongation. *Mol. Plant* 8 822–825. 10.1016/j.molp.2015.02.008 25721730

[B19] Irannejad R. Wedegaertner P. B. (2010). Regulation of constitutive cargo transport from the *trans*-Golgi network to plasma membrane by Golgi-localized G protein βγ subunits. *J. Biol. Chem.* 285 32393–32404. 10.1074/jbc.M110.154963 20720014 PMC2952241

[B20] Jiang J. Zhang C. Wang X. (2015). A recently evolved isoform of the transcription factor BES1 promotes brassinosteroid signaling and development in *Arabidopsis thaliana*. *Plant Cell* 27 361–374. 10.1105/tpc.114.133678 25649439 PMC4456931

[B21] Jones A. M. Assmann S. M. (2004). Plants: the latest model system for G-protein research. *EMBO Rep.* 5 572–578. 10.1038/sj.embor.7400174 15170476 PMC1299082

[B22] Khan S. M. Sleno R. Gora S. Zylbergold P. Laverdure J. P. Labbe J. C. (2013). The expanding roles of Gβγ subunits in G protein-coupled receptor signaling and drug action. *Pharmacol. Rev.* 65 545–577. 10.1124/pr.111.005603 23406670

[B23] Kim B. Jeong Y. J. Corvalan C. Fujioka S. Cho S. Park T. (2014). Darkness and gulliver2/phyB mutation decrease the abundance of phosphorylated BZR1 to activate brassinosteroid signaling in Arabidopsis. *Plant J.* 77 737–747. 10.1111/tpj.12423 24387668 PMC4282538

[B24] Kim T. W. Guan S. Burlingame A. L. Wang Z. Y. (2011). The CDG1 kinase mediates brassinosteroid signal transduction from BRI1 receptor kinase to BSU1 phosphatase and GSK3-like kinase BIN2. *Mol. Cell* 43 561–571. 10.1016/j.molcel.2011.05.037 21855796 PMC3206214

[B25] Kim T. W. Guan S. Sun Y. Deng Z. Tang W. Shang J. X. (2009). Brassinosteroid signal transduction from cell-surface receptor kinases to nuclear transcription factors. *Nat. Cell Biol.* 11 1254–1260. 10.1038/ncb1970 19734888 PMC2910619

[B26] Kim T. W. Wang Z. Y. (2010). Brassinosteroid signal transduction from receptor kinases to transcription factors. *Annu. Rev. Plant Biol.* 61 681–704. 10.1146/annurev.arplant.043008.092057 20192752

[B27] Kinoshita T. Cano-Delgado A. Seto H. Hiranuma S. Fujioka S. Yoshida S. (2005). Binding of brassinosteroids to the extracellular domain of plant receptor kinase BRI1. *Nature* 433 167–171. 10.1038/nature03227 15650741

[B28] Klein S. Reuveni H. Levitzki A. (2000). Signal transduction by a nondissociable heterotrimeric yeast G protein. *Proc. Natl. Acad. Sci. U.S.A.* 97 3219–3223. 10.1073/pnas.97.7.3219 10725354 PMC16219

[B29] Layden B. T. Saengsawang W. Donati R. J. Yang S. Mulhearn D. C. Johnson M. E. (2008). Structural model of a complex between the heterotrimeric G protein, Gsα, and tubulin. *Biochim. Biophys. Acta* 1783 964–973. 10.1016/j.bbamcr.2008.02.017 18373982 PMC2453506

[B30] Li J. Chory J. (1997). A putative leucine-rich repeat receptor kinase involved in brassinosteroid signal transduction. *Cell* 90 929–938. 10.1016/S0092-8674(00)80357-8 9298904

[B31] Li J. Li G. Gao S. Martinez C. He G. Zhou Z. (2010). *Arabidopsis* transcription factor ELONGATED HYPOCOTYL5 plays a role in the feedback regulation of phytochrome A signaling. *Plant Cell* 22 3634–3649. 10.1105/tpc.110.075788 21097709 PMC3015127

[B32] Li J. Nam K. H. (2002). Regulation of brassinosteroid signaling by a GSK3/SHAGGY-like kinase. *Science* 295 1299–1301. 11847343 10.1126/science.1065769

[B33] Lian H. L. He S. B. Zhang Y. C. Zhu D. M. Zhang J. Y. Jia K. P. (2011). Blue-light-dependent interaction of cryptochrome 1 with SPA1 defines a dynamic signaling mechanism. *Genes Dev.* 25 1023–1028. 10.1101/gad.2025111 21511872 PMC3093117

[B34] Mangeldorf D. J. Thummel C. Beato M. Herrlich P. Schütz G. Umesono K. (1995). The nuclear receptor superfamily the second decade. *Cell* 83 835–839. 10.1016/0092-8674(95)90199-X 8521507 PMC6159888

[B35] Mora-Garcia S. Vert G. Yin Y. Cano-Delgado A. Cheong H. Chory J. (2004). Nuclear protein phosphatases with Kelch-repeat domains modulate the response to brassinosteroids in *Arabidopsis*. *Genes Dev.* 18 448–460. 10.1101/gad.1174204 14977918 PMC359398

[B36] Oh E. Zhu J. Y. Wang Z. Y. (2012). Interaction between BZR1 and PIF4 integrates brassinosteroid and environmental responses. *Nat. Cell Biol.* 14 802–809. 10.1038/ncb2545 22820378 PMC3703456

[B37] Popova J. S. Rasenick M. M. (2003). G beta gamma mediates the interplay between tubulin dimers and microtubules in the modulation of Gq signaling. *J. Biol. Chem.* 278 34299–34308. 10.1074/jbc.M301748200 12807915

[B38] Ryu H. Kim K. Cho H. Park J. Choe S. Hwang I. (2007). Nucleocytoplasmic shuttling of BZR1 mediated by phosphorylation is essential in *Arabidopsis* brassinosteroid signaling. *Plant Cell* 19 2749–2762. 10.1105/tpc.107.053728 17873094 PMC2048706

[B39] Sang Y. Li Q. H. Rubio V. Zhang Y. C. Mao J. Deng X. W. (2005). N-terminal domain-mediated homodimerization is required for photoreceptor activity of Arabidopsis CRYPTOCHROME 1. *Plant Cell* 17 1569–1584. 10.1105/tpc.104.029645 15805487 PMC1091775

[B40] Santner A. Estelle M. (2009). Recent advances and emerging trends in plant hormone signalling. *Nature* 459 1071–1078. 10.1038/nature08122 19553990

[B41] Sprang S. R. (1997). G protein mechanisms: insights from structural analysis. *Annu. Rev. Biochem* 66 639–678. 10.1146/annurev.biochem.66.1.639 9242920

[B42] Sun Y. Fan X. Y. Cao D. M. Tang W. He K. Zhu J. Y. (2010). Integration of brassinosteroid signal transduction with the transcription network for plant growth regulation in Arabidopsis. *Dev. Cell* 19 765–777. 10.1016/j.devcel.2010.10.010 21074725 PMC3018842

[B43] Tang W. Yuan M. Wang R. Yang Y. Wang C. Oses-Prieto J. A. (2011). PP2A activates brassinosteroid-responsive gene expression and plant growth by dephosphorylating BZR1. *Nat. Cell Biol.* 13 124–131. 10.1038/ncb2151 21258370 PMC3077550

[B44] Tsugama D. Liu S. Takano T. (2013). *Arabidopsis* heterotrimeric G protein β subunit, AGB1, regulates brassinosteroid signalling independently of BZR1. *J. Exp. Bot.* 64 3213–3223. 10.1093/jxb/ert159 23814276 PMC3733146

[B45] Ullah H. Chen J. G. Temple B. Boyes D. C. Alonso J. M. Davis K. R. (2003). The beta-subunit of the Arabidopsis G protein negatively regulates auxin-induced cell division and affects multiple developmental processes. *Plant Cell* 15 393–409. 10.1105/tpc.006148 12566580 PMC141209

[B46] Ullah H. Chen J. G. Wang S. Jones A. M. (2002). Role of a heterotrimeric G protein in regulation of Arabidopsis seed germination. *Plant Physiol.* 129 897–907. 10.1104/pp.005017 12068128 PMC161710

[B47] Ullah H. Chen J. G. Young J. C. Im K. H. Sussman M. R. Jones A. M. (2001). Modulation of cell proliferation by heterotrimeric G protein in *Arabidopsis*. *Science* 292 2066–2069. 10.1126/science.1059040 11408654

[B48] Urano D. Chen J. G. Botella J. R. Jones A. M. (2013). Heterotrimeric G protein signalling in the plant kingdom. *Open Biol.* 3:120186 10.1098/rsob.12.0186 PMC3718340 23536550

[B49] Urano D. Jones A. M. (2014). Heterotrimeric G protein-coupled signaling in plants. *Annu. Rev. Plant Biol.* 65 365–384. 10.1146/annurev-arplant-050213-040133 24313842 PMC4861148

[B50] Vert G. Nemhauser J. L. Geldner N. Hong F. Chory J. (2005). Molecular mechanisms of steroid hormone signaling in plants. *Annu. Rev. Cell Dev. Biol.* 21 177–201. 10.1146/annurev.cellbio.21.090704.151241 16212492

[B51] Wang W. X. Lian H. L. Zhang L. D. Mao Z. L. Li X. M. Xu F. (2016). Transcriptome analyses reveal the involvement of both C and N termini of cryptochrome 1 in its regulation of phytohormone-responsive gene expression in *Arabidopsis*. *Front. Plant Sci.* 7:294. 10.3389/fpls.2016.00294 27014317 PMC4789503

[B52] Wang X. Q. Ullah H. Jones A. M. Assmann S. M. (2001). G protein regulation of ion channels and abscisic acid signaling in *Arabidopsis* guard cells. *Science* 292 2070–2072. 10.1126/science.1059046 11408655

[B53] Wang Y. Sun S. Zhu W. Jia K. Yang H. Wang X. (2013). Strigolactone/MAX2-induced degradation of brassinosteroid transcriptional effector BES1 regulates shoot branching. *Dev. Cell* 27 681–688. 10.1016/j.devcel.2013.11.010 24369836

[B54] Wang Z. Y. Bai M. Y. Oh E. Zhu J. Y. (2012). Brassinosteroid signaling network and regulation of photomorphogenesis. *Annu. Rev. Genet.* 46 701–724. 10.1146/annurev-genet-102209-163450 23020777

[B55] Wang Z. Y. Nakano T. Gendron J. He J. X. Chen M. Vafeados D. (2002). Nuclear-localized BZR1 mediates brassinosteroid-induced growth and feedback suppression of brassinosteroid biosynthesis. *Dev. Cell* 2 505–513. 10.1016/S1534-5807(02)00153-3 11970900

[B56] Xu D. B. Gao S. Q. Ma Y. N. Wang X. T. Feng L. Li L. C. (2017). The G-Protein beta subunit AGB1 promotes hypocotyl elongation through inhibiting transcription activation function of BBX21 in Arabidopsis. *Mol. Plant* 10 1206–1223. 10.1016/j.molp.2017.08.004 28827171

[B57] Yamamoto R. Fujioka S. Iwamoto K. Demura T. Takatsuto S. Yoshida S. (2007). Co-regulation of brassinosteroid biosynthesis-related genes during xylem cell differentiation. *Plant Cell Physiol.* 48 74–83. 10.1093/pcp/pcl039 17132633

[B58] Yang H. Q. Tang R. H. Cashmore A. R. (2001). The signaling mechanism of Arabidopsis CRY1 involves direct interaction with COP1. *Plant Cell* 13 2573–2587. 10.1105/tpc.13.12.2573 11752373 PMC139474

[B59] Yang H. Q. Wu Y. J. Tang R. H. Liu D. Liu Y. Cashmore A. R. (2000). The C termini of Arabidopsis cryptochromes mediate a constitutive light response. *Cell* 103 815–827. 10.1016/S0092-8674(00)00184-7 11114337

[B60] Yang M. Li C. Cai Z. Hu Y. Nolan T. Yu F. (2017). SINAT E3 ligases control the light-mediated stability of the brassinosteroid-activated transcription factor BES1 in Arabidopsis. *Dev. Cell* 41 e44. 10.1016/j.devcel.2017.03.014 28399399 PMC6283279

[B61] Yin Y. Vafeados D. Tao Y. Yoshida S. Asami T. Chory J. (2005). A new class of transcription factors mediates brassinosteroid-regulated gene expression in *Arabidopsis*. *Cell* 120 249–259. 10.1016/j.cell.2004.11.044 15680330

[B62] Yin Y. Wang Z. Y. Mora-Garcia S. Li J. Yoshida S. Asami T. (2002). BES1 accumulates in the nucleus in response to regulate gene expression and promote stem elongation. *Cell* 109 181–191. 10.1016/S0092-8674(02)00721-3 12007405

[B63] Yu T. Y. Shi D. Q. Jia P. F. Tang J. Li H. J. Liu J. (2016). The Arabidopsis receptor kinase ZAR1 is required for zygote asymmetric division and its daughter cell fate. *PLOS Genet.* 12:e1005933. 10.1371/journal.pgen.1005933 27014878 PMC4807781

[B64] Zhang J. Liu W. Liu J. Xiao W. Liu L. Jiang C. (2010). G-protein β2 subunit interacts with mitofusin 1 to regulate mitochondrial fusion. *Nat. Commun.* 1:101. 10.1038/ncomms1099 20981029

[B65] Zhang J. Y. He S. B. Li L. Yang H. Q. (2014). Auxin inhibits stomatal development through MONOPTEROS repression of a mobile peptide gene STOMAGEN in mesophyll. *Proc. Natl. Acad. Sci. U.S.A.* 111 E3015–E3023. 10.1073/pnas.1400542111 25002510 PMC4115497

[B66] Zhang Y. Li C. Zhang J. Wang J. Yang J. Lv Y. (2017). Dissection of HY5/HYH expression in *Arabidopsis* reveals a root-autonomous HY5-mediated photomorphogenic pathway. *PLOS ONE* 12:e0180449. 10.1371/journal.pone.0180449 28683099 PMC5500333

