# Corrigendum: Arabidopsis G-Protein β Subunit AGB1 Interacts With BES1 to Regulate Brassinosteroid Signaling and Cell Elongation

**DOI:** 10.3389/fpls.2020.01122

**Published:** 2020-07-22

**Authors:** Ting Zhang, Pengbo Xu, Wenxiu Wang, Sheng Wang, Julie C. Caruana, Hong-Quan Yang, Hongli Lian

**Affiliations:** ^1^ School of Life Sciences and Biotechnology, Shanghai Jiao Tong University, Shanghai, China; ^2^ State Key Laboratory of Genetic Engineering and Collaborative Innovation Center for Genetics and Development, Institute of Plant Biology, School of Life Sciences, Fudan University, Shanghai, China; ^3^ Department of Cell Biology and Molecular Genetics, University of Maryland, College Park, MD, United States; ^4^ Key Laboratory of Urban Agriculture (South), School of Agriculture and Biology, Shanghai Jiao Tong University, Shanghai, China

**Keywords:** BR signaling pathway, G-protein signaling pathway, BES1, AGB1, protein interaction, phosphorylation status, transcription activity

In the original article, there was a mistake in **Figure 1B** as published. In our original data, there were six different yeast cells in each pair, and the high similarity among them caused an error in capturing images. The corrected **Figure 1B** appears below.

The authors apologize for this error and state that this does not change the scientific conclusions of the article in any way. The original article has been updated.

**Figure 1:**
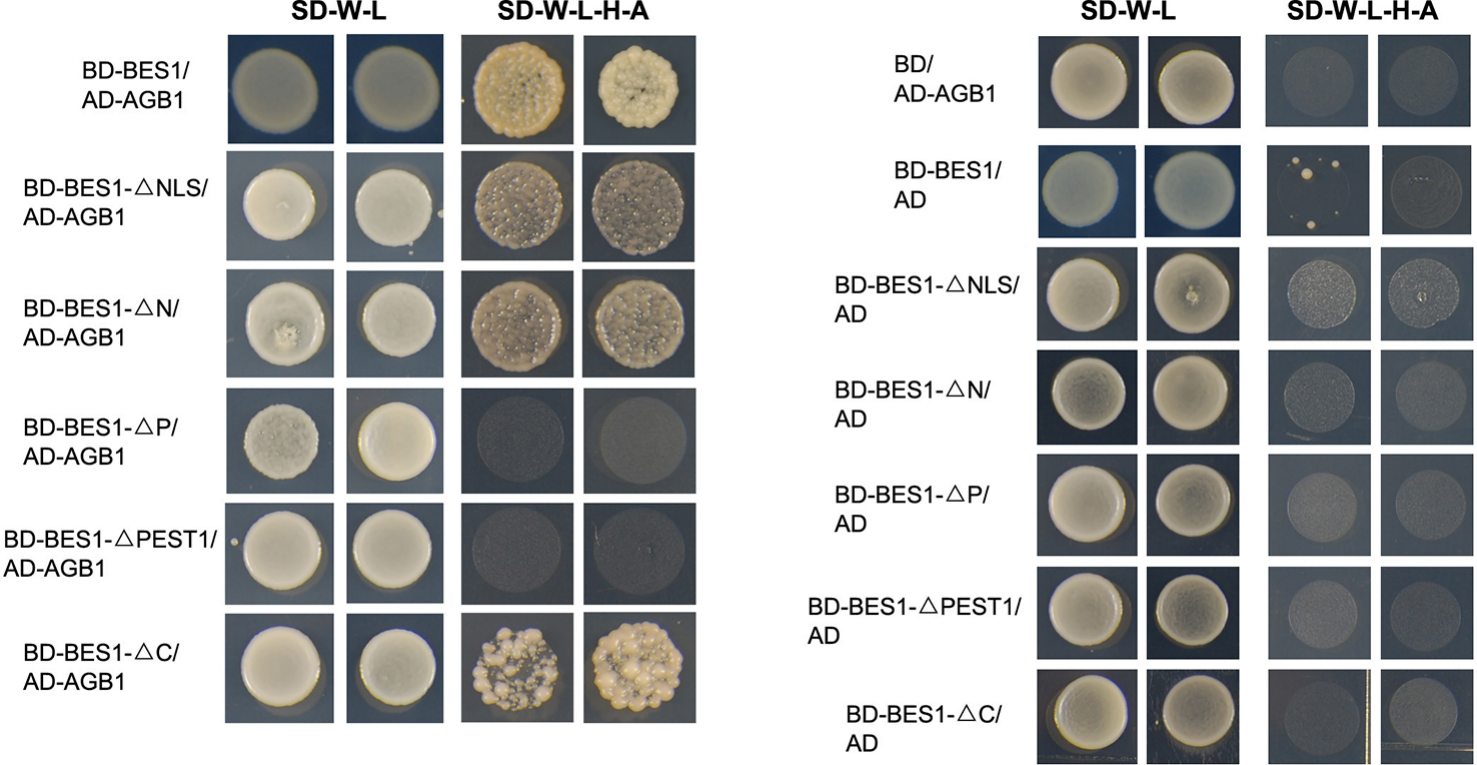
AGB1 interacts with BES1 *in vitro*. **(A)** Schematic structure of various BES1 fragments fused to the GAL4 DNA-binding domain (BD) for yeast two-hybrid assays. **(B)** The P and PEST domain of BES1 are necessary and sufficient for the interaction of AGB1 with BES1. Yeast cells co-expressing the indicated combinations of constructs were grown on basic (SD-T-L) or selective (SD-T-L-H-A) media for 3-6 days **(C)** AGB1 interacts with BES1 by pull down assay *in vitro*. GST-BES1 pulls down His-AGB1, but not His-TF (negative control). AGB1 interaction with BES1 was detected with anti-His antibody.

