# Supplementary material for: Arabidopsis G-Protein β Subunit AGB1 Interacts with BES1 to Regulate Brassinosteroid Signaling and Cell Elongation
Source: Front Plant Sci. 2018 Jan 9;8:2225. doi: 10.3389/fpls.2017.02225 (PMC5767185; doi:10.3389/fpls.2017.02225)
Supplement: Supplementary file 1 [file Data_Sheet_1.PDF]

***Arabidopsis* G-protein β subunit AGB1 interacts with BES1 to regulate brassinosteroid signaling and cell elongation**

Ting Zhang, Pengbo Xu, Wenxiu Wang, Sheng Wang, Hongquan Yang and Hongli Lian*

Supplementary data

Supplementary table 1 List of primers used for plasmid construction.

Assays	Constructs	Primer name	Primer sequence (5'→3')
Yeast-two hybrid	pGBKT7-BES1	BES1-EcoRI-F	GGAATTCCATGACGTCTGACGGAGCAACGT
		BES1-SmaI-R	CCCCGGGGTCAACTATGAGCTTTACCATTT
	pGBKT7-BES1-∆NLS (∆21-41)	BES1-EcoRI-F	GGAATTCCATGACGTCTGACGGAGCAACGT
		BES1-∆NLS-60-R	GTATAAATCTTCGCCGCAACCGTCGCCATCGCTGCT
		BES1-∆NLS-124-F	AGCAGCGATGGCGACGGTTGCGGCGAAGATTTATAC
		BES1-SmaI-R	CCCCGGGGTCAACTATGAGCTTTACCATTT
	pGBKT7-BES1-∆N (∆42-98)	BES1-EcoRI-F	GGAATTCCATGACGTCTGACGGAGCAACGT
		BES1-∆N-123-R	GAGTTGCTCGAGAAGATGATCCAGCTCTTCTCCGCCGCTCTC
		BES1-∆N-295-F	GAGAGCGGCGGAGAAGAGCTGGATCATCTTCTCGAGCAACTC
		BES1-SmaI-R	CCCCGGGGTCAACTATGAGCTTTACCATTT
	pGBKT7-BES1-∆P (∆99-196)	BES1-EcoRI-F	GGAATTCCATGACGTCTGACGGAGCAACGT
		BES1-∆P-294-R	GCCATGGACATGGATTGTTTAGCCATGTCACCAGGTAGAG
		BES1-∆P-589-F	CTCTACCTGGTGACATGGCTAAACAATCCATGTCCATGGC
		BES1-SmaI-R	CCCCGGGGTCAACTATGAGCTTTACCATTT
	pGBKT7-BES1-∆PEST (∆231-250)	BES1-EcoRI-F	GGAATTCCATGACGTCTGACGGAGCAACGT
		BES1-∆PEST-690-R	CTTTTGAAAGCTTATCCAATGGAACTGGCGATGATGAGTAG
		BES1-∆PEST-751- F	CTACTCATCATCGCCAGTTCCATTGGATAAGCTTTCAAAAG
		BES1-SmaI-R	CCCCGGGGTCAACTATGAGCTTTACCATTT
	pGBKT7-BES1-∆C (∆268-335)	BES1-EcoRI-F	GGAATTCCATGACGTCTGACGGAGCAACGT
		BES1-∆C-804-ter BglII-R	GAAGATCTTCAAGAGGCAGAGAATGGCTGTTGT

	pGADT7-AGB1	AGB1-EcoRI-F	CGGAATTCATGTCTGTCTCCGAGCTCAAAGAAC
		AGB1-XhoI-R	CCCCTCGAGTCAAATCACTCTCCTGTGTCCTCCA
	pLexA-AGB1	AGB1-EcoRI-F	CGGAATTCATGTCTGTCTCCGAGCTCAAAGAAC
		AGB1-XhoI-R	CCCCTCGAGTCAAATCACTCTCCTGTGTCCTCCA
	pB42AD-BIM1	BIM1-MfeI-F	GGCCAATTGATGGAGCTTCCTCAACCTCGTC
		BIM1-XhoI-R	CCCCTCGAGCTACTGTCCCGTCTTGAGCCGTTT
Pull-down	pCold-TF-AGB1	AGB1-EcoRI-F	CGGAATTCATGTCTGTCTCCGAGCTCAAAGAAC
		AGB1-XhoI-R	CCCCTCGAGTCAAATCACTCTCCTGTGTCCTCCA
	pGEX4T-1-BES1	BES1-EcoRI-F	GGAATTCCATGACGTCTGACGGAGCAACGT
		BES1-SmaI-R	CCCCGGGGTCAACTATGAGCTTTACCATTT
Semi-in *vivo* pull down	pGEX4T-1-AGB1	AGB1-EcoRI-F	CGGAATTCATGTCTGTCTCCGAGCTCAAAGAAC
		AGB1-XhoI-R	CCCCTCGAGTCAAATCACTCTCCTGTGTCCTCCA
	*35S::*BES1-GFP-Flag	infusion BES1-GFP-Flag-F	TCTCTCTCTCAAGCTTATGACGTCTGACGGAGCAACG
		infusion BES1-GFP-Flag-R	TCGAGGATCCAAGCTTTTAATGGCCCGGGCTAGCATGCAT
Co-IP	*35S::*mBES1-GFP-Flag	infusion BES1-GFP-Flag-F	TCTCTCTCTCAAGCTTATGACGTCTGACGGAGCAACG
		BES1-M-R	CACATTCAGGTATAGTAGCCGGAGCATGGAACTGGCGATG
		BES1-M-F	CATCGCCAGTTCCATGCTCCGGCTACTATACCTGAATGTG
		infusion BES1-GFP-Flag-R	TCGAGGATCCAAGCTTTTAATGGCCCGGGCTAGCATGCAT
	*35S::*AGB1-MYC-HA	AGB1-SalI-F	GGGGTCGACATGTCTGTCTCCGAGCTCAAAGAAC
		AGB1-BamHI-R	GCCGGATCCTCAAATCACTCTCCTGTGTCCTCCA
Yeast-one hybrid	pLaci-DWF4	DWF4-EcoRI-F	CGGAATTCGGGTTTGACTGTCCAGTTCGGTAAT
		DWF4-XhoI-R	CCCCTCGAGACCCTTAGGATATGGGAAAAGGGTG

	pB42AD-BES1	infusion-BES1-F	AGCCTCTCCCGAATTATGACGTCTGACGGAGCAACGT
		infusion-BES1-R	TCCAAAGCTTCTCGAGTCAACTATGAGCTTTACCATTT
Dual-LUC	pGreenII 0800-DWF4$_{pro}$-LUC	DWF4$_{pro}$-2000-XhoI-F	CCCCTCGAGATATACATAATTCCTTACAGAA
		DWF4$_{pro}$-1-AvrII-R	GCCCCTAGGGGAGCTAGTTTCTCTCTCTCTC
	35S::BES1-Flag	BES1-BamH1-F	CGGATCCGATGACGTCTGACGGAGCAAC
		BES1-Spe1-R	GACTAGTCACTATGAGCTTTACCATTTCCA
	PHB-AGB1-NLS-YFP	AGB1-BamH1-F	GCCGGATCCATGTCTGTCTCCGAGCTCAAAGAAC
		AGB1-Spe1-R	GGACTAGTTCAAATCACTCTCCTGTGTCCTCCA
Protein Colocalization	PHB-BIM1-CFP	BIM1-BamHI F	GCCGGATCCATGGAGCTTCCTCAACCTCGC
		BIM1-ter-SpeI R	GGACTAGTCTACTGTCCCGTCTTGAGCCGTTT

Supplementary table 2 List of primers used for quantitative RT-PCR.

Gene	Forward primer (5'→3')	Reverse primer (5'→3')
PP2A	TATCGGATGACGATTCTTCGTGCG	GCTTGGTCGACTATCGGAATGAGG
CPD	TTGCTCAACTCAAGGAAGAG	TGATGTTAGCCACTCGTAGC
DWF4	GGTGATCTCAGCCGTACATTTGA	CCCCACGTCGAAAAACTACCACTC
IAA19	GGTGACAACTGCGAATACGTTAA	CCCGGTAGCATCCGATCTTTTCA
EXP16	CGCTCTCATTTCGACCTCGCCAT	CGTTCGTAATCAGCACCAAGTT
SAUR15	AAGAGGATTCATGGCGGTCTATG	GTATTGTTAAGCCGCCCATTGG
SAUR-AC1	GCTTAACAATACCATGTCATGAATCTTT	TCTGAGATGTGACTGTGAAGAACAA

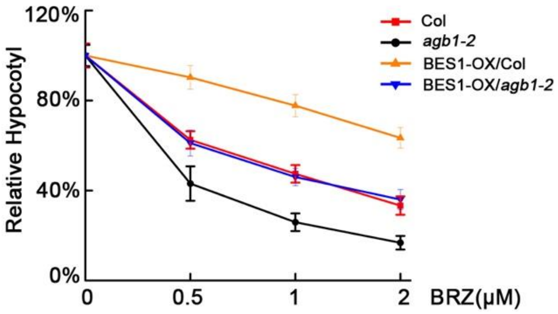

Figure S1 Hypocotyl lengths of BES1-Flag-overexpressing plants grown in the presence of BRZ. Col, *agb1-2*, BES1-Flag/Col and BES1-Flag/*agb1-2* in the presence of 0 μM, 0.5 μM, 1 μM and 2 μM BRZ. Seedlings were grown in the darkness for 5 days. Values are means ± SD. Error bars represent SD (n＞25) and the hypocotyl lengths of seedlings in the absence of BRZ were defined as "100%".

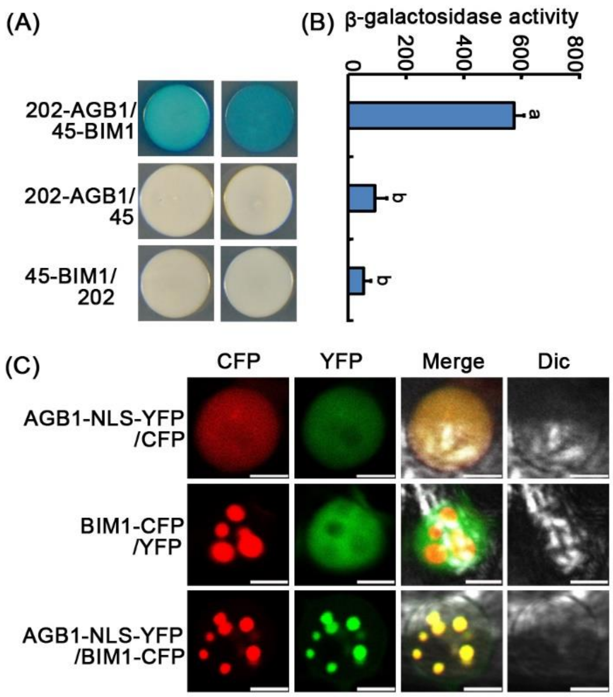

Figure S2 The interaction between AGB1 and BIM1 *in vitro* and *in vivo*.

(A) Yeast two-hybrid assay showing the interaction of AGB1with BIM1.

(B) Quantitative yeast two-hybrid assay for **(A)**. The letters "a" to "b" denote statistically significant differences between the indicated samples, as determined by Tukey's LSD test ($P \leq 0.01$). Error bars represent means ± SD ($n = 6$).

(C) Colocalization study in tobacco cells showing AGB1 and BIM1 colocalize to the same nuclear bodies in tobacco cells. Scale bar = 5 μm.

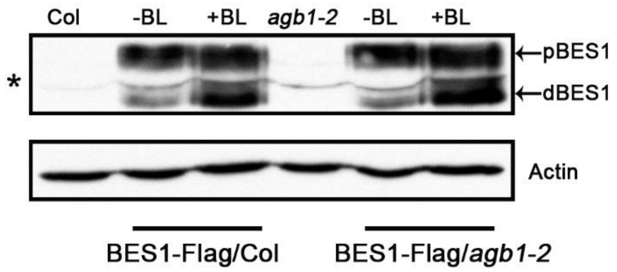

Figure S3 Expression levels of the transgenic plants BES1-Flag/Col and BES1-Flag/*agb1-2* in presence of BL. The transgenic plants BES1-Flag/Col and BES1-Flag/*agb1-2* were grown in the presence of 20 nM BL for 15 days (+ BL), or in the absence of BL (-BL), and used for Western blotting using an anti-Flag antibody. The upper arrow denotes pBES1, the lower arrow denotes dBES1. Asterisk denotes non-specific band.

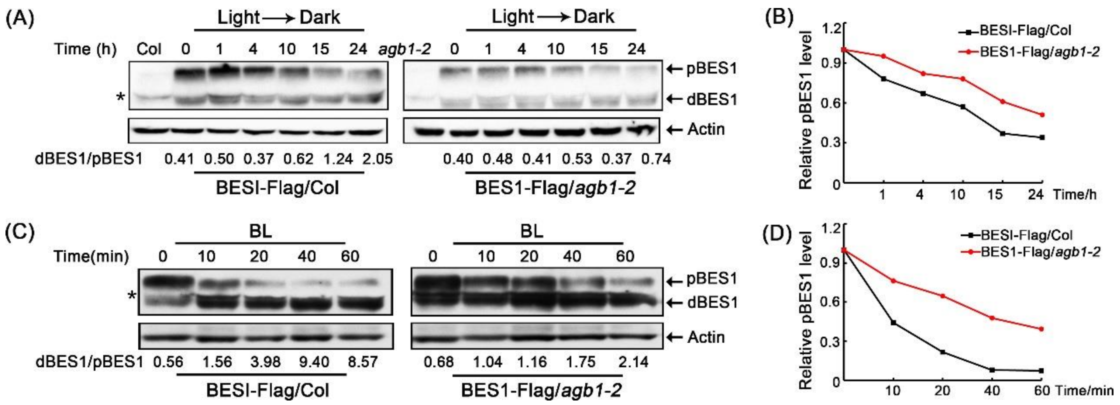

Figure S4 The pBES1 protein degraded faster in *BES1-Flag*/Col than in *BES1-Flag*/*agb1-2* under darkness and BL treatment.

(A) and **(C)** Western blot analysis of phosphorylation status of BES1 in Col or *agb1-2* plants expressing BES1-Flag in darkness **(A)** and under BL treatment **(B)**. Six-day -old BES1-Flag transgenic seedlings were kept in darkness or treated with BL for the indicated periods of time and used to prepare protein to detect the dephosphorylated BES1 (dBES1) and phosphorylated BES1 (pBES1) with anti-Flag antibody. Actin was used as a loading control. Data indicates the ratio of dBES1/pBES1. The upper arrow denotes pBES1, the lower arrow denotes dBES1. Asterisk denotes non-specific band.

(B) and **(D)** Analysis of pBES1 protein level in Col or *agb1-2* plants expressing BES1-Flag in darkness **(A)** and under BL treatment **(C).** The pBES1 protein level in the absence of darkness **(A)** and BL **(C)** were defined as “1.0”.

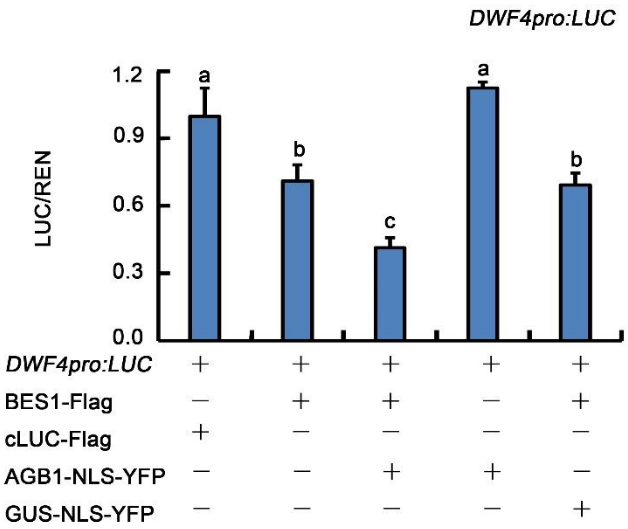

Figure S5 AGB1 promotes the transcription level of BES1 in tobacco leaves. Tobacco leaves were infiltrated with strains harboring the *DWF4pro: LUC* reporter and effectors in the indicated combinations. cLUC-Flag and GUS-NLS-YFP served as negative controls for BES-Flag and AGB1-NLS-YFP, respectively. Expression values are determined by calculating the ratio of LUC activity to REN activity (LUC/REN). The letters “a” to “c” denote statistically significant differences between the indicated samples, as determined by Tukey’s LSD test ($P \leq 0.05$). Error bars represent SD ($n = 3$). The LUC/REN of the combination of *DWF4pro::LUC* and cLUC-Flag were defined as “1.0”.
